# The Impact of Educational Attainment on Black Women’s Obesity Rate in the United States

**DOI:** 10.1007/s40615-019-00663-z

**Published:** 2019-11-14

**Authors:** Gwenetta Denise Curry

**Affiliations:** 1 University of Edinburgh, Edinburgh, UK; 2 The University of Alabama, 101 Manly Hall, Tuscaloosa, AL 35487 USA

**Keywords:** Health disparities, Race, Obesity, Gender, Education

## Abstract

Over the last two decades, Black women have been disproportionately impacted by the obesity epidemic in the USA. According to the Centers for Disease Control and Prevention (CDC), 56.6% of Black women are overweight or obese compared with 44.4% Hispanic and 32.8% of white women. Social scientists and public health researchers have argued that increasing educational attainment would lead to overall improvements in health outcomes. Using the National Health and Nutrition Examination Survey (NHANES), a nationally representative cross-sectional survey, Cycles 1999–2010, I examined how educational attainment impacts Black women’s rate of obesity and C-reactive protein levels (*N* = 2685). Multiple linear regression was used to analyze the association between body mass index (BMI) and educational attainment. C-reactive protein, inflammation response, was used to measure the body’s reaction to being exposed to stress. The results demonstrated that educational attainment among Black women does not decrease their risk of being obese or levels of C-reactive protein. This article provides evidence to support a need to increase awareness of health disparities that disproportionately impact Black women.

**Electronic supplementary material:**

The online version of this article (10.1007/s40615-019-00663-z) contains supplementary material, which is available to authorized users.

## Introduction

Over the last decade, obesity has become a major pandemic on a global scale driven by local markets and changes in food distribution [26, 43]. According to the Center for Disease Control and Prevention (CDC), 39.8% of American adults are considered obese and affect 93.3 million US adults in 2015–2016 [9]. Healthy People 2020 has set a target to reduce the number of adults who are considered obese to 30% by the year 2020 [12]. Ogden and others found that among men, 27.4% with a college degree are obese compared with 32.1% of those with less than a high school education, although these differences were not statistically significant [31]. However, there is a trend showing that women with college degrees were less likely to be obese than those without a college degree [31]. National data indicate that the obesity epidemic disproportionately impacts Black women compared with their male and white male and female counterparts [24, 29, 35]. The research on the contributing factors of obesity among Black women has been inconsistent [4]. Black women’s economic disadvantages impact their access to resources, and stress levels have a negative impact on their health outcomes [9, 34]. While there are many contributing factors to the condition of Black women, studies no longer deny the impact that one’s environment has on health outcomes. Black women’s biological response to stress plays a major role in obesity rates and obesity-related illnesses in this group [16, 37]. Elevated concentrations of C-reactive protein have been seen in adults with exposure to stress associated with socioeconomic disadvantages [7, 28, 32], and there is a confirmed link between CRP and obesity [5, 15, 28, 41]. More research is needed to evaluate the impact education has on obesity rates among Black women and the influence of stress on this relationship.

This study aims to examine the association of educational attainment, C-reactive protein, and obesity among Black women in the USA. Understanding the factors that affect obesity risk among Black women is important because four out of every five Black women are overweight or obese [7], and there are serious health-related consequences for individuals and their families, including the development of cardiometabolic disease(s) and lower quality of life. Black women have higher levels of degree attainment than their Black male counterparts [21, 19]; it is critical to understand the relationship between education and obesity.

### Education and Health

The idea that years of higher levels of education might protect against obesity is generally consistent with a larger body of research on socioeconomic status and health. For example, several studies have uncovered an inverse relationship between socioeconomic status and various indicators of physical health [1, 2, 17, 29]. Although education is sometimes included in composite measures of socioeconomic status, researchers have not begun to understand the unique ways that higher levels of education influence obesity among Black women. Previous research has shown that there is a relationship between psychosocial stressors, such as racism, to health outcomes among Blacks [3, 5]. It remains clear that prolonged exposure to stress has an impact on health outcomes, and Geronimus’ [16] research supports this claim by showing that Black women aged more rapidly than whites due to stress. Black women’s subjective experience of racism—the stigmas others have of them—can pose serious health threats and appear to be related to obesity and weight gain. Despite these well-established facts, medical sociologists continue to focus on race as only a demographic measure without considering the multiple effects impact that racial inequality has on the overall health outcomes of Blacks.

Previous research by Holmes et al. [20, 22, 23] focused on educational differences in obesity among Blacks, as a part of their larger study of the links between educational attainment and obesity across racial/ethnic groups. These investigators argued that educational attainment reduces the rate of obesity through several contributing factors. These factors include differences in income, occupation, and access to resources that are necessary to live a healthy lifestyle. Holmes and Zajacova used the National Health Interview Survey 1997–2012 to determine if whites experience greater benefits from educational attainment than other minorities [19]. They found that higher educational attainment affects the health of whites more than minorities, even when potential sociodemographic, behavioral, and economic mediators are considered.

Supporting research by Zhang and Wang demonstrated that while obesity increased in all race-sex groups from 1971 to 2000, white women had a clear inverse association between obesity and educational attainment over time, and white men in the low socioeconomic status group experienced a decrease in obesity from 1999 to 2002 [42]. In the same study, among Black women, the association between obesity and education switched from an inverse relationship to the medium SES group having the highest prevalence of obesity by 1999. Black women at every educational level are disproportionately affected by the obesity epidemic compared with their white counterparts [20]. Jackson et al. found that Black women with greater than a high school education had substantially higher BMIs than white women with less than a high school education [21].

According to the National Health and Nutrition Examination Survey (NHANES) data from 1976 to 2004, African-American women ages 20 to 74 obesity rates increased from 15.6 to 20% when compared with white women over time [14]. The longitudinal data on the development of obesity indicates that the rise in obesity rates in women has been the greatest in African-American women, followed by Hispanic women, then white women [10]. Obesity was significantly more prevalent among Black men as of NHANES II but by a much smaller margin than women in the survey [14].

With over half the Black women in America being considered overweight or obese [10, 23], scholars must consider the possibility that some psychosocial factors are impacting Black women differently than other populations. Among men, there was little difference in the prevalence of obesity by race/ethnicity [18]. Among women, however, the overall 1999–2010 prevalence among non-Hispanic Black (51%) was 10% higher than that among Mexican-Americans and 20 percentage points higher than that among non-Hispanic whites [18]. According to the CDC, “Black women with a bachelor’s degree had a median income of $33, 877, which was 111 percent of the $30, 413 median income figures for non-Hispanic white women who held a college degree. It is clear then that the strong income performance of Black colleges is largely due to the earnings performance of Black women, while higher education has failed to produce similar income gains for Black men in comparison to white men [19].” Although these figures show Black women with bachelor’s degrees making slightly more money than white women with the same education, it does not tell the complete story. Compared with white women, Black women possessed less access to education, had high-paying occupations, and were less able to maximize those resources given the societal structure [25, 26]. Fewer resources and larger amounts of debt contribute to the difficulty Black women have with upward mobility [5]. The lack of social mobility among Black women increases their proximity to poverty and decreases their access to healthy foods and safe places to exercise [5, 39]. In combination, these conditions make Black women more vulnerable to negative health outcomes.

Previous studies have shown that individuals with lower socioeconomic status have high levels of stress and were at greater risk of obesity compared with individuals with high socioeconomic status [9, 27]. Recently, Black women have become the most educated population in the USA [36], but their health outcomes have not followed the same trajectory. There are mediating factors that need to be investigated to gain a clear understanding of what is preventing Black women from having better health outcomes with higher socioeconomic status.

## Methods

### Dataset

The National Health and Nutrition Examination Survey (NHANES) is a program of studies designed to assess the health and nutritional status of adults and children in the US beginning in 1960 [15]. NHANES is a major program of the National Center for Health Statistics as a part of the Centers for Disease Control and Prevention and has the responsibility for producing vital and health statistics for the Nation. “The survey combines an in-home interview and a standardized physical examination at a mobile examination center (MEC). In each survey, a nationally representative sample of the US civilian noninstitutionalized population was selected using a complex, stratified, multistage probability cluster sampling design [7].”

Beginning in 1999, NHANES became a continuous survey without a break between cycles to focus on a variety of health and nutrition measurements to meet emerging needs [7]. The procedures followed in selecting the sample and conducting the interview and examination were similar to those for previous surveys. Household interviews and physical examinations were conducted for each survey participant. During the physical examination, conducted in mobile examination centers, height and weight were measured as part of a more comprehensive set of body measurements [11]. These measures were taken by trained health technicians, using standardized measuring procedures and equipment. The NHANES examines a nationally representative sample of roughly 5000 persons each year [11]. The participants were in counties across the country, 15 of which are visited every year.

### Participants

This study will focus on Black women age 20–84 who participated in the Continuous NHANES from the years 1999–2010. Observations for pregnant women and women missing a valid height or weight measurements were excluded from the data analysis. For the current research, the final NHANES analytical sample consisted of 3488 Black women over the age of 20 years.

## Measurements

### Dependent Measures

#### Body Mass Index and Obesity

Participants’ weight and height were measured in a mobile examination center using standardized techniques and equipment. Body mass index (BMI) was expressed as weight in kilograms divided by the square of height in meters. Adults are commonly classified as overweight (BMI 25.0–29.9), obese (BMI greater than or equal to 30.0), and extreme obesity (BMI greater than or equal to 40) among adults (ages 20 years and over). Body mass index will be used to measure obesity in three different ways: continuous, dichotomous (0 = not obese, 1 = obese), and categorical (1 = underweight, 2 = normal, 3 = overweight, 4 = obese).

### Independent Measures

#### Educational attainment

Education will be used to measure socioeconomic status (SES) and has been suggested by many scholars to be the most stable and robust indicator of SES. Other SES variables, such as income and occupation, vary over time and have a lower response rate than education items. Educational attainment will be measured using the question “What is the highest grade or level of school completed or the highest degree received?” Education was coded as a categorical variable where 1 = less than high school, 2 = high school, 3 = some college or associate, and 4 = college graduate or greater. The reference group will be those with “high school” education. Education will be treated as an independent variable rather than a moderator when testing the hypotheses_._

#### Serum C-reactive protein

Participants’ serum C-reactive protein measures were taken in the mobile examination center with values ranging from 0.01 to 18.01 mg/dL. C-reactive protein measures inflammation response, and participants with levels greater than or equal to 0.3 indicate high risk. Analysis will be measured in two ways: continuous and dichotomous (0 = < 0.3; 1 = > 0.3).

### Control Measures

#### Food Insecurity

Household food security status was based on the NHANES household food security category designation, which was determined using the 18-item US Food Security Survey Module known as the “Core food security module.” Only one respondent per household answered the module, even if more than one family is living in a household. Screening into the module depended on responses to previous questions, and those households screened out were assumed to have negative responses to all module questions. Households that give uniformly negative responses to the early questions will have a very low likelihood of having experienced any conditions of food insecurity and can safely be deemed to be food secure [6]. The two levels of screening are as follows: The first-level screen, including optional Q1. “We always have enough to eat and the kinds of food we want” and respond “never true” (or “don’t know” or “refuse”) to all five of the questions Q2 to Q6 [10]. Second-level screening for households not previously screened out responded “never true” (or “don’t know” or “refuse”) to “The children were not eating enough because we just out couldn’t afford enough food” was omitted. Food security question referred to circumstances over the 12 months preceding the survey. The NHANES categories are as follows: “fully food secure,” “marginally food secure,” “food insecure without hunger,” and “food insecure with hunger.” Participants were considered “fully food secure” if they had no affirmative response in any items from the food module. Participants who were considered “marginally food secure” answered affirmatively to 1 or 2 items in the module. The participants considered having “low food security” answered affirmatively to 3–5 items in the module. The participants who were “very low food security” answered 6 to 10 items in the module in the affirmative.

#### Hours Worked

Hours worked was measured through a series of questions related to the number of hours worked at all their jobs or businesses in the last week (continuous numbers).

#### Physical Activity

Physical activity was measured with items concerning daily activity and activity over the last 30 days. If they did not participate in vigorous or moderate activity, their response was coded as 0 = no and 1 = yes if they exercised. Responses of “Unable to do activity,” “Refused,” and “Don’t Know,” were coded as missing.

#### Food Measures

Twenty-four-hour dietary recall interviews were conducted where foods and beverages consumed the previous 24 h ending at midnight were solicited and recorded using the standardized Automated Multiple Pass Method (AMPM) [29]. “With the exception of a few entry screens, all of the Dietary Recall section of the interview was collected using the AMPM program and all of the post-recall session is collected using the wrapper program. The 24hr dietary recall collects a list of all the foods and beverages consumed within a 24hr period; the time of consumption and the name of the eating occasion; detailed food descriptions and amounts of the reported foods; where it was obtained; and whether it was eaten at home or not. The recall data is coded and linked to the database of foods and their nutrient composition. Calculations of total daily nutrient intakes were derived from these data” [10].

Additionally, food measure guidelines were given to participants for assistance in estimating portion sizes during the interview. Fruit intake will be measured using a dummy variable (0 = no fruit, 1 = fruit). Vegetable intake will be measured using a dummy variable (0 = no vegetable, 1 = vegetable).

#### Income

Participants were asked to disclose information regarding their total annual family income. National Center for Health Statistics used the US Bureau of Census Current Population Survey definition of “family” to household group members into one or more families. The CPS defines a family as “a group of two people or more (one of whom is the householder) related by birth, marriage, or adoption and residing together; all such people (including related subfamily members) are considered to be members of one family” [7]. Total family income reported as a range of values in dollars was measured on a 15-point scale (1 = < 4999 and 15 = > 100,000). The income groups were combined to generate three income categories (1 = < 20,000, 2 = 21,000–54,999, and 3 = 55,000 to > 100,000).

#### Age

Participants were asked, “Best age in years of the sample person at the time of HH screening. Individuals 80 and over were top coded at 80 years of age [11].” Age will be broken down into three different groups 1 = 20–39, 2 = 40–59, and 3 = 60–80. Age was also measured as a continuous variable.

#### Marital Status

Marital status measured as a (0 = not married and1 = married/living with a partner). The not married category will include divorced, widowed, separated, and never married. Participants who responded “refused” or answered “don’t know” were coded as missing. The “Married” category will be the reference group.

### Statistical Analysis

Data will be analyzed using Stata version 13.1, College Station, Texas, for Windows software programs. All analyses included sample weights that account for the unequal probabilities of selection, oversampling, and nonresponse. To account for the complex survey design survey set (SVYSET), primary sampling unit (PSU) and stratified sampling (STRATA) codes were used for unequal probabilities of sample selection in NHANES.

Descriptive statistics were calculated for sociodemographic characteristics, food insecurity and C-reactive protein, waist measurements, body mass index, and weighted with the sample weights calculated for the 12-year sample in NHANES. Multiple linear regressions will be used to analyze the association between BMI, waist, and education. Also, some of the regression models will contain interaction terms to test whether education moderates the effect of C-reactive protein on obesity.

To test for multicollinearity, I ran the variance inflation factor analysis and found all the results to be below 10. Indicating there was not a problem with variance inflations.

## Results and Discussion

### Descriptive Analysis

Table 1 is a descriptive table that shows the makeup of the participants in the dataset. The mean/percentages and standard deviations for the overall sample of Black women by education group—less than high school, high school, some college and associate, and college. Most of the Black women in the study had some college or associate degree education 32.4% compared with 31.1, 22.6, and 13.9% for less than high school, high school, and college, respectively. The overall average age is 47.1 years old. The average age across education groups is 36.4, 41.2, 45.1, and 44.9 for the less than high school, high school, some college and associate, and college educated, respectively.

**Table 1:** Descriptive statistics

	Education category				
	Overall	Less than HS	High school/GED	Some college or associate	College graduate
Less than high school	31.1%				
High school/GED	22.6%				
Some college or associate	32.4%				
College graduate	13.9%				
BMI (kg/m**2) (ave)	31.1	30.2	31.3	34.0	30.2
Waist circumference (cm)	97.0	94.3	96.9	102.0	95.3
BMI category					
Underweight	0.8%	1.2%	0.8%	0.6%	0.7%
Normal weight	21.0%	21.8%	23.1%	21.8%	26.9%
Overweight	27.4%	30.1%	26.2%	21.2%	26.1%
Obese	50.85	46.9%	50.0%	56.5%	46.3%
CRP (%)					
Low risk	46.2%	43.4%	44.1%	48.8%	50.7%
High risk	53.7%	56.6%	55.9%	51.3%	49.3%
Age (ave years)	47.1	36.4	41.2	45.1	44.9
Work hours (ave)	22.8	13.9	22.9	25.5	32.8
Work hours (%)					
< 40 h	65.4%	80.9%	64.2%	58.4%	47.3%
> 40 h	34.6%	19.1%	35.8%	41.6%	52.7%
< 55 h	92.5%	97.2%	92.8%	91.4%	84.0%
> 55 h	7.47%	2.8%	7.2%	8.7%	16.0%
Marital status (%)					
Not married	63.8%	68.9%	63.8%	62.6%	55.5%
Married/cohabitating	36.3%	31.1%	36.2%	37.4%	44.5%
Income (%)					
< $20,000	39.9%	54.4%	38.6%	35.9%	14.3%
$20,000 to $54,000	42.8%	33.4%	46.7%	46.2%	46.6%
> $55,000	17.3%	12.2%	14.7%	17.9%	39.2%
Physical activity (%)					
No	58.6%	71.8%	59.2%	48.8%	52.3%
Yes	41.4%	28.2%	40.9%	51.2%	47.7%
Food security (%)					
Fully food secure	68.5%	63.8%	64.5%	69.5%	83.5%
Marginally food secure	13.5%	13.9%	15.8%	13.7%	8.3%
Food insecure w/o hunger	10.6%	13.1%	11.2%	10.2%	5.1%
Food insecure w/ hunger	7.3%	9.2%	8.5%	6.6%	2.9%
Fruit intake					
No	91.8%	91.1%	89.7%	93.1%	93.5%
Yes	8.2%	8.9%	10.3%	6.9%	6.5%
Vegetable intake					
No	78.1%	79.6%	80.5%	75.8%	76.3%
Yes	21.9%	20.4%	19.5%	24.2%	23.7%
Number of observations	*N* = 2658	*N* = 826	*N* = 600	*N* = 862	*N* = 370

The average overall body mass index was 31.1 kg/m*2, and across education groups, the numbers exceed 30.0; 30.2, 31.3, 34.0, and 30.2 kg/m*2 for less than high school, high school, some college, and college, respectively. These results demonstrate that for all levels of education, the average BMI is in the obese range. The some college and associate education group had the highest average BMI, which is very different from what other studies have found with white populations. According to the NHANES Survey from 2005 to 2008, white women with less than high school education had the highest rate of obesity (42.1%) compared with 21.8% with a college degree [30].

The results show that overall 50.8% of Black women in this study are considered to be obese, and 27.4% are overweight. Black women with some college and associate degrees had the highest percentage of obese women, with 56.5% being categorized as obese compared with 46.9, 50.0, and 46.3% for less than high school, high school, and college, respectively. College-educated Black women had the highest percentage of women who were considered “normal” weight, 26.9% compared with 21.8, 23.1, and 21.8% for less than high school, high school, and some college and associate, respectively.

The overall waist circumference for Black women was 97.0 cm. Across education groups, the average waist circumference was 94.3, 96.9, 102.0, and 95.3 cm for less than high school, high school, some college and associate, and college, respectively. Similar to what was seen with the average BMI scores, waist circumference was the highest for the some college and associate education groups. Forty percent of Black women in this study participated in some moderate or vigorous physical activity in the past 30 days. Black women with the some college and associate degrees had the highest participation in physical activity with 51.2% participation compared with 28.3%, 40.9%, and 47.7% for less than high school, high school, and college, respectively. C-reactive protein levels decreased as the level of education increased but remained in the high-risk range after obtaining a college degree. Black women with less than a high school education had the highest C-reactive protein level 0.70 compared with .66, .57, and 0.53 for the high school, some college or associate, and college degree, respectively. The majority of the participants had a high-risk C-reactive protein level, 53.7%, compared with 46.2%. Black women with a college degree had the lowest percentage with high-risk C-reactive protein levels, 49.3 compared with 56.6, 55.9, and 51.3 for less than high school, high school, and some college or associate, respectively.

Majority of the population worked less than 40 h a week (65%), and those with less than a high school education had the highest percentage working under 40 h, 80.9% compared with 64.2, 58.4, 47.3 for high school, some college or associate, and college educated, respectively. Black women with a college degree had the highest percentage of working more than 55 h (15%) and 40 h a week (52%). Interestingly, work hours increased as the level of education increased, demonstrating that education does not protect Black women from working long hours.

The majority of the Black women in this study made between 20,000 and 54,000 dollars in the last year. As expected, Black women with less than a high school education had the highest percentage of women making less than 20,000 dollars in the last year. Also, Black women with college degrees had the highest percentage of women making more than 50,000. The percentage of women making between 20,000 and 54,000 dollars was practically the same for the high school, some college and associate, and college-educated women, 46.7%, 46.2%, and 46.6%, respectively.

Overall, 36.3% of the Black women were either married or cohabitating with a partner. Across education groups, the majority of the women were not married or cohabitating, and the women with less than high school education had the highest percentage not married, 68.9% compared with 63.8%, 62.6%, and 55.5% for high school, some college or associate, and college educated, respectively. The college-educated women had the highest percentage of marriage 44.5% compared with 31.1%, 36.2%, and 37.4% for the less than high school, high school, and some college and associate, respectively.

Fruit and vegetable consumption was rather low across all groups. The lowest fruit consumption was found among the population with less than high school population, 91.1% did not consume any fruit during the 24-h dietary recall. The high school–educated women had the highest percentage of fruit consumption 10.3% compared with 8.9%, 6.9%, and 6.5% for the less than high school, some college and associate, and college educated, respectively. Vegetable consumption was much higher than fruit consumption. Overall, 21.9% consumed vegetables during the 24-h dietary recall. Black women with some college or associate degrees had the highest consumption of vegetables 24.2% compared with 20.4%, 19.5%, and 23.7% for the less than high school, high school, and college educated, respectively.

The majority of the population was considered to be fully food secure with college-educated Black women having the highest percentage of participants who were fully food secure, 83.5% compared with 63.8%, 64.5%, and 69.5% for the less than high school, high school, and some college and associate, respectively. Overall, only 7.3% of the total group was considered to be food insecure with hunger.

Table 2 contains six different models measuring variables associated with body mass index of Black women. Model one indicates that relative to Black women with a college degree, women with some college education were more likely to have a higher body mass index. Model 2 shows that C-reactive protein is positively associated with body mass index with a *p* value of 0.00. Model 3 includes education and C-reactive protein. The results show when education and C-reactive protein are in the model together, the women with some college and associate education remained statistically different from those with college education. Also, high-risk C-reactive protein continued to be significantly different from low-risk C-reactive protein in a positive direction. In Model 4, the control variables married, age, and cycle intake were all added to the equation along with C-reactive protein (a measure of stress). Model 5 included food security, fruit and vegetable, and physical activity. Model 6 added the education, C-reactive protein variable.

**Table 2:** Linear regression body mass index of black women in the National Health and Nutrition Examination Survey Cycles 1999–2010

	Model 1	Model 2	Model 3	Model 4	Model 5	Model 6
	Education b/se	CRP b/se	Education and CRP b/se	Control b/se	Food b/se	Interaction b/se
Less than high school	0.998		0.402	3.168**	0.341	0.875
	(− 0.58)		(− 0.55)	(− 1.08)	(− 0.58)	(− 0.6)
High school	0.95		0.446	2.843**	0.358	0.419
	(− 0.57)		(− 0.55)	(− 1.05)	(− 0.59)	(− 0.59)
Some college or associate	1.483**		1.171*	3.124**	1.039	1.257*
	(− 0.56)		(− 0.54)	(−1.05)	(− 0.56)	(−0.56)
High-risk CRP		7.103***	7.100***	14.694***	7.094***	7.591***
		(− 0.34)	(− 0.34)	(−0.68)	(− 0.35)	(− 0.74)
Married/cohabitating				0.366	− 0.06	− 0.072
				(− 0.62)	(− 0.32)	(− 0.32)
Age				0.103***	0.012	0.012
				(− 0.02)	(− 0.01)	(− 0.01)
NHANES cycle				0.674***	0.198	0.201
				(− 0.2)	(− 0.1)	(− 0.1)
Vegetable intake					− 0.24	− 0.246
					(− 0.43)	(−0.43)
Fruit intake					0.536	0.543
					(− 0.51)	(−0.51)
Physical activity					− 0.285	− 0.275
					(− 0.31)	(− 0.3)
Food security						
Marginally food secure					0.302	0.293
					(−0.36)	(− 0.36)
Food insecure w/o hunger					1.641**	1.648**
					(− 0.58)	(− 0.58)
Food insecure with hunger					1.987**	1.970**
					(−0.6)	(− 0.61)
Work hours					0.018*	0.018*
					(− 0.01)	(− 0.01)
Less than high school X CRP						− 1.058
						(− 0.95)
High school/ GED X CRP						− 0.186
						(− 0.84)
Some college or associate X CRP						− 0.48
						(− 0.92)
Constant	30.649***	27.957***	27.342***	82.732***	25.722***	25.500***
	(− 0.46)	(− 0.18)	(− 0.42)	(− 1.35)	(− 0.72)	(− 0.76)
Observation	2,597	2,597	2,597	2,564	2,564	2,564
*R*^2^	0.003	0.177	0.18	0.206	0.189	0.19
Degrees of freedom	90	90	90	89	89	89

Standard errors in parenthesis

***p < 0.00, **p < 0.01, *p < 0.05

Body mass index for Black women with some college or associate education continued to differ significantly from those having a college degree. The results show that age was significantly associated with body mass index. Also, serum C-reactive protein level > 0.3 mg/dl (high risk) was significantly associated with body mass index with a *p* value < 0.01. Stated another way, exposure to stress has a significant positive impact on body mass index compared with the reference group of C-reactive protein level < 0.3 (low risk).

Model 5 included all of the measures for the previous four models with the addition of food security, fruit and vegetable intake, and physical activity. Participants with some college or associate degree were not significantly associated with body mass index. Compared with the reference category of fully food secure, being marginally food secure was not associated with body mass index (*p* value < 0.01). There was a statistical difference between the reference groups and “food insecure w/ hunger” and “food insecure w/o hunger.” Participants being physically active and eating fruits and vegetables were not statically different from the reference categories. The findings indicate that these positive health behaviors did not have an association with body mass index. These results do not imply that healthy behaviors (physical activity and fruit and vegetable consumption) could not potentially have a positive impact on maintaining healthy body weight. Instead, these results confirm the finding of previous work by Cozier et al. [13] demonstrating that more efforts should be made to understand how psychosocial factors (e.g., stress, experiences of racism, sexism, and discrimination) impact health outcomes in Black women. Said differently, while exercise and healthy lifestyle habits should be recommended for Black women, these findings suggest that stress must also be considered a factor increasing BMI and decreasing the benefits of exercise and diet.

Model 6 included all the measures for the previous five models with the addition of interaction variables. Compared with the reference category, Black women with some college or associate degree was significantly associated with body mass index (*p* value < 0.01). There was no statistical difference between education and C-reactive protein interaction. Food insecurity with hunger and with hunger were both statistically significant. Compared with the reference group working long hours was significantly associated with BMI. These results mirror that of other research findings showing long work hours increase not only stress levels but also reduce the time one has for physical activity and healthy eating [26, 27].

In summary, other studies found there to be a gradient decrease in body mass index and the risk of obesity as education increases [20, 23, 38]. Educational attainment has been linked directly to health outcomes among white populations [40, 33]. The current data challenges this view by demonstrating there is not a gradient decrease in obesity and body mass index with educational attainment among Black women. Black women with some college and associate education were more likely to be overweight and had a higher risk of obesity compared with women with a college degree.

Also, these results support the previous literature by Brooks et al. [4, 7, 8, 38], demonstrating that C-reactive protein has a strong association with body mass index and increases the odds of being obese when levels are > 0.3 mg/dL for Black women.

## Conclusion

While other populations’ obesity rates have remained steady over the years, Black women’s rate has increased and continues to outnumber their white female and male counterparts. Educational attainment has long been touted as a solution to decreasing negative health outcomes. This research challenges the current understanding of the relationship between educational attainment and obesity. My research sought to (1) analyze the obesity trends among Black women from 1999–2010 by education and age, (2) gain a better understanding of the psychosocial factors contributing to Black women’s risk of obesity across levels of education, and (3) research the impact of stress on Black women’s risk of obesity.

In the current study, Black women with some college or associate degree were more likely to be overweight or obese compared with other education groups. While the causes of this increased risk are unclear, one possible explanation is women with some college education are working multiple jobs, which can result in limited time for food preparation and exercise. Cozier’s [13] study found a strong association between experiences of discrimination and obesity rates. While the current study did not measure discrimination and racism, stress was measured using serum C-reactive protein levels. In line with other research, body mass index and being obese was strongly associated with C-reactive protein levels equal to or greater than 0.03 mg/dl. Waist circumference also had a strong positive association with C-reactive protein levels. Numerous possible factors could contribute to Black women’s stress levels, but that data was not available for the study. However, the research performed by others demonstrates that Black women experience racism and discrimination. Perceived discrimination based on race and gender is a key factor in chronic stress-related health disparities among ethnic/racial and other minority groups [19, 30, 33]. Cozier’s research suggests workplace and community-based programs to combat racism and interventions to reduce racism-related stress could be important components of strategies for the prevention of obesity [13]. Her findings also suggest that experiences of racism possibly explain why the obesity epidemic disproportionately impacts US Black women. Other studies have linked maternal stress as a potential explanation for excess preterm delivery among Black women because of exposure to racism associated stress [13].

## Broader Implications

Being overweight or obese can lead to several life-threatening diseases and a lower quality of life. According to the CDC, obesity has been linked to increased risk of hypertension, diabetes, high cholesterol, stroke, and some cancers. High rates of obesity will have an overall increase in the cost of medical care and disability services provided by federal funds.

## Electronic Supplementary Material

ESM 1 (DOCX 28 kb)
