# Supplementary material for: The Impact of Educational Attainment on Black Women’s Obesity Rate in the United States
Source: J Racial Ethn Health Disparities. 2019 Nov 14;7(2):345–54. doi: 10.1007/s40615-019-00663-z (PMC7064624; doi:10.1007/s40615-019-00663-z)
Supplement: Supplementary file 1 — (DOCX 28 kb) [file 40615_2019_663_MOESM1_ESM.docx]

Supplemental Data

**Matrix of correlations**

| Variables | (1) | (2) | (3) | (4) | (5) | (6) | (7) | (8) | (9) |
| --- | --- | --- | --- | --- | --- | --- | --- | --- | --- |
| (1) BMI | 1.000 |  |  |  |  |  |  |  |  |
| (2) Age | -0.044 | 1.000 |  |  |  |  |  |  |  |
| (3) Educ | 0.066 | -0.251 | 1.000 |  |  |  |  |  |  |
| (4) CRP | 0.409 | 0.015 | 0.025 | 1.000 |  |  |  |  |  |
| (5) Physical Act | -0.022 | -0.092 | 0.269 | -0.052 | 1.000 |  |  |  |  |
| (6) Fruit and Veg | 0.009 | 0.012 | 0.044 | -0.014 | 0.030 | 1.000 |  |  |  |
| (7) Food Security | -0.010 | -0.132 | -0.021 | -0.004 | 0.049 | -0.032 | 1.000 |  |  |
| (8) Married | -0.067 | -0.047 | 0.063 | -0.135 | 0.033 | 0.014 | -0.099 | 1.000 |  |
| (9) Smoker | -0.057 | 0.061 | -0.030 | 0.008 | 0.007 | -0.011 | 0.111 | -0.139 | 1.000 |
|  | | | | | | | | | |

**Variance inflation factor**

|  | VIF | 1/VIF |
| --- | --- | --- |
| education | 1.881 | .532 |
| C-Reactive Pro | 5.812 | .172 |
| Age | 1.177 | .849 |
| Married | 1.13 | .885 |
| smoker | 1.107 | .903 |
| Family Inc | 1.248 | .801 |
| Alcohol | 1.12 | .893 |
| Physical Act | 1.08 | .926 |
| Fruit and Veg | 1.008 | .992 |
| Food Security | 1.106 | .904 |
| 2.Educ#c.CRP | 2.977 | .336 |
| 3.Educ#c.CRP | 5.144 | .194 |
| 4.Educ#c.CRP | 2.435 | .411 |
| Mean VIF | 2.094 | . |
